# Brief cognitive behavioral therapy in pregnant women at risk of postpartum depression: Pre-post therapy study in a city in southern Brazil

**DOI:** 10.1016/j.jad.2021.04.031

**Published:** 2021-07-01

**Authors:** Ricardo Tavares Pinheiro, Jéssica Puchalski Trettim, Mariana Bonati de Matos, Karen Amaral Tavares Pinheiro, Ricardo Azevedo da Silva, Clarissa Ribeiro Martins, Gabriela Kurz da Cunha, Fernanda Teixeira Coelho, Janaína Vieira dos Santos Motta, Fábio Monteiro da Cunha Coelho, Gabriele Ghisleni, Fernanda Nedel, Ana Paula Ardais, Rafaelle Stark Stigger, Luciana de Avila Quevedo, Luciano Dias de Mattos Souza

**Affiliations:** a Catholic University of Pelotas, Brazil; b Federal University of Rio Grande, Brazil; c Federal University of Pelotas, Brazil; d PQ CNPq, Brazil

**Keywords:** Pregnancy, Prevention, Postpartum depression, Cognitive behavior therapy

## Abstract

**Background:**

Postpartum depression (PPD) affects a high number of women, often the first manifestation of a mood disorder that will occur later in life, bringing serious consequences for the patient and her offspring. Depression today is the leading cause of disability worldwide. The aim of this study was to evaluate the effectiveness of a preventive cognitive behavioral therapy (CBT) for PPD.

**Methods:**

Pre-post therapy study, as part of a population-based cohort study. Pregnant women without a diagnosis of depression participated, who were divided into two groups: risk of depression (CBT) and a control group (without therapy). The preventive therapy consisted of six sessions of CBT, administered weekly. The Outcome Questionnaire (OQ-45) was used in all sessions. The Mini International Neuropsychiatric Interview and Beck Depression Inventory-II were used on three occasions. The final statistical analyses were performed by Poisson regression.

**Results:**

The prevalence of PPD in the risk group was 5.5% and in the control group 2.2%, with no difference between the groups (PR 1.66 95% CI 0.44-6.18). The OQ-45 averages gradually reduced during the therapy sessions, indicating therapeutic progress. Schooling was an associated factor, both with the manifestation of PPD and with the greater effectiveness of the therapy.

**Limitations:**

Rate of 40.5% refusal to preventive treatment and absence of a group with similar characteristics in another therapy model.

**Conclusions:**

Brief cognitive behavioral therapy applied by mental health professionals with basic training was effective in preventing the manifestation of PPD.

## Highlights

• Brief CBT in pregnancy was effective in preventing PPD.
• CBT improved interpersonal relationships and understanding of social status (OQ-45).
• Education, among the risk factors for PPD, was decisive in the effectiveness of CBT.

## Introduction

Pregnancy and postpartum are periods characterized by physical, emotional, social and hormonal changes that make women more susceptible to the increase in, or appearance of some symptoms or even full-blown psychiatric disorders, such as depression (Hartmann et al., 2017). Depression during the pregnancy-puerperal cycle presents rates that can vary between 10 and 20% (Becker et al., 2016; Pereira and Lovisi, 2008) for the pregnancy period and between 15 and 25% (Moraes et al., 2006) in the postpartum period. The incidence of postpartum depression (PPD) is 12% (Shorey et al., 2018). As a result of this work there is an increased effort by researchers to help define an effective, low-cost and, especially a preventive treatment for the puerperal period. Therefore minimizing the traumatic consequences that inevitably impact the whole family.

The literature points to some factors associated with PPD, such as low social support, lack of family planning, presence of anxiety, low levels of schooling and history of past depression (Beck et al., 2016; Matsumura et al., 2019; Pereira and Lovisi, 2008). The treatments are effective for gestational and postpartum depression (Huang et al., 2018; Stephens et al., 2016), however most studies still work with treatment therapies, whereas descriptions of effectiveness of preventive models of the disease are rare.

Therefore, we are looking for psychotherapeutic therapy models that can prevent the emergence of this condition, with brief therapy protocols and consequently, a lower cost to healthcare (Trivedi, 2014). In this context, cognitive-behavioral therapies (CBT) have gained prominence, because based on behavioral and cognitive changes, they present evidence of improvement of depressive symptoms and functionality of individuals through cognitive restructuring and behavioral activation techniques (Beck, 2019; Huang et al., 2018). The CBT is a model that has been shown to be significantly efficient for depressed patients (Okumura and Ichikura, 2014; Santoft et al., 2019) and also for the prevention of depression in risk and/or subsyndromal cases (Allart-van Dam et al., 2007; O'Connor et al., 2016).

Considering that depression is the leading cause of disability worldwide, research on the efficacy of preventive protocols for PPD can collaborate with important information about the overall burden of the disease (Saúde, 2021). Maternal depression, specifically, generates high costs to health systems, which could be minimized if preventive treatments of the disease were implemented.

The aim of this study was to evaluate the effectiveness of a preventive cognitive behavioral therapy for depression in the postnatal period in women at risk of developing PPD. As secondary aims, therapeutic progress was investigated during the sessions of preventive psychotherapy as well as the sociodemographic and health factors associated with PPD.

## Methods

### Outline

This is a pre-post therapy study, part of a population-based cohort study.

### Sample capture

The sampling process began in 2016 and was carried out in multiple stages, with census tracts derestricted by the Brazilian Institute of Statistics (IBGE) as initial sampling units. Census tracts are territorial units for conducting census operations with defined criteria, in continuous areas and respecting the political-administrative divisions of Brazil. These are relatively homogeneous units in terms of population characteristics, economic status, living conditions and number of inhabitants. First, the 488 census tracts of the urban area of Pelotas, a city in southern Brazil, were listed, according to the 2010 Census (IBGE 2010) for random sampling selection of 244 tracts (50.0% of the total of the urban area). Subsequently, each of the tracts drawn received a visit from the capture team, and all pregnant women were identified. Those who were up to 24 weeks pregnant were invited to participate in the study. The capture lasted 24 months. The tracts were covered twice and all the houses were visited. The first visit in the first 12 months and the second in the last year of capture. This strategy was established on an estimated basis to obtain a sample power of 80% for the PPD outcome in pregnant women without depression. The sample size calculation for this study was done using the parameters of the prevalence of postpartum depression of 20%, power of 80% and margin of error of two percentage points. With an increase of 30% for losses and refusals, 514 individuals would be needed. However, the calculation that led to the need to capture a higher number of pregnant women concerns other hypotheses linked to other objectives that required a larger sample size.

The follow-up evaluations occurred at three time periods: between the first and second gestational trimesters (T1 - pre-therapy), performed in the participants' homes; the second, performed in an outpatient setting between 60 and 90 days after the first evaluation (T2 - post-therapy); and the third 90 days after delivery (T3) when the PPD outcome was evaluated.

#### General inclusion criteria

All women up to 24 weeks of gestation, residing in the randomized census tracts were invited to participate. Pregnant women who agreed to sign the Informed Consent Form, who did not have a diagnosis of the current Major Depressive Episode (Mini Plus) and consented to participate in therapy if necessary, were included. Thus, pregnant women from the urban area of the city of Pelotas were selected who agreed to carry out all the steps proposed in the study.

#### General exclusion criteria

Pregnant women diagnosed with major depression and/or with a moderate or severe suicide risk, assessed by Mini Plus at T1 were excluded from the analyses, as well as women who were already undergoing psychotherapeutic or pharmacological treatment for mental disorders elsewhere and dependent on substances (except tobacco).

#### Eligibility criteria for the risk group for PPD (therapy by CBT)

For inclusion in the risk group for depression, it was necessary to present at least three of the following criteria: unplanned current pregnancy; presence of psychiatric disorders in the nuclear family; less than 9 years of schooling; presence of depressive and/or anxious subsyndromal symptoms; presence of 4 or more stressful events in the last year; presence of chronic disease; absence of a partner; absence of mother support and presence of a major depressive episode in the past.

#### Eligibility criteria for control group (no therapy)

The control group was composed of pregnant women who, in addition to not being diagnosed with depression, did not present a risk for depression (see previous section), and the other exclusion criteria described above (see section “General exclusion criteria”). This group did not receive any type of therapy, but they were evaluated during the follow-ups.

#### Preventive cognitive behavioral psychotherapy

The CBT technique offered, consisted of an adapted version of the manual of Cognitive Behavioral Psychotherapy (Beck and Miller, 2021; Beck, 2011; Beck, 1997), structured according to the proposal made by Aaron Beck (Beck, 1964). This model proposed psychotherapy in six sessions that addressed distorted and/or dysfunctional thoughts (which influence the patient's mood and behavior) focused on the pregnancy-puerperal cycle. Eligible participants received weekly sessions of 50 minutes of individual psychotherapy, totaling 6 sessions. The first session was focused on establishing therapeutic alliance and identifying dysfunctionalities in cognition, emotion and/or behavior that were related to potential depressive symptoms. The main objective of the second session was the psychoeducation of the cognitive model and the understanding of the role of thoughts in the dysfunctionalities identified in the first session. Still in the second session, as a homework assignment, an exercise of self-monitoring was prescribed. The third session aimed to improve the self-monitoring technique and the understanding of how to apply the cognitive model in the life of the pregnant woman. Cognitive and/or behavioral techniques were used in the fourth and fifth sessions to improve patients coping strategies. In the last session, the entire process was revised in order to reinforce the skills learned.

Psychotherapy was performed at the Psychological Clinic of the Catholic University of Pelotas (UCPel) and was supervised by a researcher with training in the proposed model. Data analysis included all pregnant women who attended at least one psychotherapy session (intention-to-treat analysis).

This study had psychotherapists (psychologists and psychiatrists) with up to five years of previous experience in the field of mental health, but without specific training in CBT, who were then trained in this procedure. The team had training based on a manual created for the proposed therapy with the purpose of reducing doubts and standardizing the sessions. Thus, the therapists had weekly meetings with the general coordinator and with the teacher responsible for the therapy for the purpose of monitoring and supervising each participant.

#### Measures

The outcome - PPD - was evaluated through the Mini International Neuropsychiatric Interview (M.I.N.I. PLUS + Brazilian Version 5.0.0), three months after delivery (T3). This interview aims at the diagnostic classification of the interviewees in a manner compatible with the criteria of the Diagnostic Manual of Mental Disorders (DSM-IV-TR) and the International Classification of Diseases (ICD 10) and the "plus" version of the instrument allows for the clinical judgment of the interviewer. In this work, the "A" module was used, which investigates the presence of a major depressive episode present or past. The same instrument was used at the time of the first evaluation with the study participants (T1), in a home interview and, likewise, in the second evaluation (T2), which took place in an outpatient setting. Suicide risk as an exclusion criteria also was assessed by module C of the interview (Amorim, 2000).

The Outcome Questionnaire (OQ-45.2) was used in all sessions to monitor the results of psychotherapy. This is a self-assessment questionnaire designed specifically for the evaluation of changes during psychotherapy treatments. It consists of 45 questions with answer options ranging from 0 (never) to 4 (always), related to psychological suffering, interpersonal relationships and the social role of the patient. Thus, the lower the score, the greater the perception of improvement of patients throughout psychotherapy (Da Silva et al., 2016).

The depressive and anxious subsyndromal symptoms were evaluated using the Beck scales. The Beck Depression Inventory (BDI-II) (Gomes-Oliveira et al., 2012) and the Beck Anxiety Inventory (BAI) (Beck et al., 1988) were applied to all follow-up evaluations. Both scales consist of 21 statements about cognitive, affective and somatic symptoms, in the case of BDI and common symptoms of anxiety and in the case of BAI. The total score of each instrument ranges from 0 to 63 points, and for this study the cut-off point of 10 to 18 points was considered as subsyndromal depression (BDI-II) and from 9 to 18 points as subsyndromal anxiety (BAI). We used the BDI-II as a continuous mean as an indicator of severity of symptoms of depression, calculating the difference of means between the times of evaluation and by group (control and risk). As well as analysis of the means adjusting for the factors associated with the outcome.

The identification of the number of stressful events in the last year was performed through the Holmes and Rahe Social Readjustment Assessment Scale (1967), composed of 24 situations in which the pregnant woman answered whether or not she experienced any of them in the last 12 months. The instrument is based on the proposition that the effort required for the individual to adjust to society, after significant changes in her life, takes such a toll that it leaves the patient prone to diseases. In this study the events were grouped into "up to 3 events" or "4 events or more" and the pregnancy event was replaced by marital reconciliation (Holmes and Rahe, 1967; Savoia, 1999).

The economic evaluation of the participants was carried out through the classification of ABEP (Brazilian Association of Research Companies), which is based on the accumulation of material goods, the schooling level of the head of the family, whether the house is located on a paved road and if it has a mains water supply. This classification groups the participants into socioeconomic levels A, B, C, D or E, from the scores achieved, and the letter "A" refers to the highest socioeconomic level and "E" to the lowest (www.abep.org). For this study, the pregnant women were categorized as follows: A+B (higher levels), C (medium level) and D+E (lower levels) (Abep, 2015).

The other variables: age (collected in years and later categorized as tertile), lives with a partner (yes/no), pregnancy planning (yes/no), presence of psychiatric disorders in the nuclear family (father, mother or siblings - yes/no), schooling (collected in full years of schooling, later dichotomized considering a division by quartile and establishing the cut-off point for the lower quartile of up to 8 years of schooling), presence of chronic disease (hypertension, diabetes, thyroid or lupus - yes/no) and support of the mother in relation to pregnancy (yes/no) were investigated through specific questions contained in the general questionnaire.

#### Data processing and analysis

The data was double-entered in EpiData 3.1 (Lauritsen, 2002) to check for inconsistencies, and later transferred to the Statistical Package for the Social Sciences (SPSS) (IBM, 2016) and Stata (Gould, 2021), where statistical analyses were performed by simple and relative frequencies, mean and standard deviation, chi-square test, t-test, ANOVA and *Poisson* regression, when the multivariate analysis was carried out, all variables with p < 0.20 were adjusted in the bivariate analysis.

The variable of the z-score of the risk criteria was created from the sum of the 10 variables of the referral checklist (see section “Eligibility criteria for the risk group for PPD”).

#### Ethical aspects

The project in which this study is linked was approved by the research ethics committee of the Catholic University of Pelotas under protocol number 47807915.4.0000.5339, reference number 1.729.653 and Trial registration Universal Trial Number (UTN) U1111-1227-9789.

## Results

**Fig. 1:**
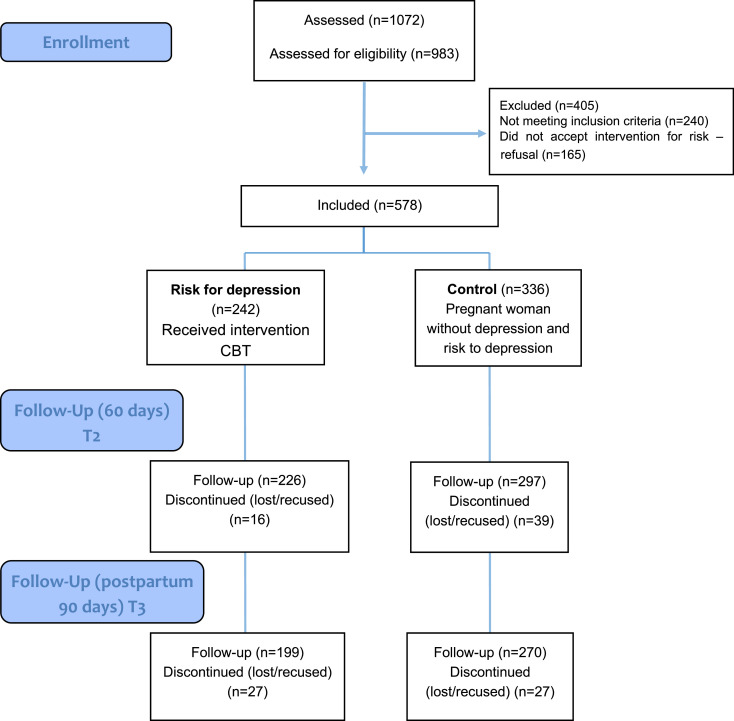
Consort flow chart in the intervention study.

In this study 578 pregnant women were included. Of that total, 336 were included in the control group, i.e., women without risk of depression and that therefore did not receive any therapy. The remaining 242 pregnant women were included in the risk group for depression and these received preventive therapy for postpartum depression using a CBT protocol. During the follow-up evaluations, both the control group and the risk group suffered drop-outs (details in Fig. 1).

Among the 242 pregnant women in the at risk group who started treatment, 199 (82.2%) participated in therapy and the follow-up assessment 90 days after delivery. In the control group, 270 women (80.3%) participated in the postpartum evaluation.

Exploratory analyzes to verify the factors associated with losses and refusals were carried out. As for the risk group, we did not find any difference in the Z score of the risk criteria for depression between the pregnant women who underwent the therapy and the pregnant women at risk who refused to participate and were excluded from this study (p = 0.855). Failure to attend the postpartum assessment was not associated with this Z score (p = 0.660) as well as with sociodemographic factors (age, living with a partner, socioeconomic status) (p >  0.05).

As for the control group, having less schooling was associated with the non-continuation of postpartum follow-up (PR 2.28 95% CI 1.23-4.23).

The first analysis of the effect of the therapy comparing the two groups (control group/no therapy and the at risk group/therapy) was performed at T2 when the mean gestational age was 27.1 weeks (SD ± 5.9). The incidence of gestational depression at this time was 0.7% for the control group and 2.2% for the risk group, with no difference between the groups (p = 0.248).

Table 1 presents the characterization of the baseline sample (N = 578) and the bivariate analysis between the exposure variables and PPD (N = 469). They were significantly associated with the outcome having up to 8 years of schooling (p = 0.017) and having presented subsyndromal depression during pregnancy (p = 0.008).

**Table 1:** Characterization of the sample and bivariate analysis through the x^2^ test between the exposure variables and PPD.

	**Characterization of the sample**			**PPDX^2^ (N = 469)**	
	**Control**	**Risk**	**Total**		
	**N (%)**	**N (%)**	**N (%)**	**N (%)**	**p-value**
Age					0.099
Up to 23 years	99 (29.5)	82 (33.9)	181 (31.3)	03 (2.2)	
Between 24 and 29 years	102 (30.4)	71 (29.3)	173 (29.9)	03 (2.1)	
30 years or more	135 (40.2)	89 (36.8)	224 (38.8)	11 (5.9)	
**Socioeconomic level**^a^^b^					0.333
A+B	116 (35.0)	61 (25.8)	177 (31.2)	04 (2.7)	
C	185 (55.9)	132 (55.9)	317 (55.9)	09 (3.5)	
D+E	30 (9.1)	43 (18.2)	73 (12.9)	04 (7.0)	
**Schooling**^a^^b^					0.009
Up to 8 years of study (lower quartile)	64 (19.1)	92 (38.0)	156 (27.0)	09 (7.9)	
9 years of study or more	271 (80.9)	150 (62.0)	421 (73.0)	08 (2.3)	
**Lives with a partner**^b^					0.085
No	23 (6.8)	45 (18.6)	68 (11.8)	04 (8.3)	
Yes	313 (93.2)	197 (81.4)	510 (88.2)	13 (3.1)	
**Family member with psychiatric illness**^b^					0.291
No	274 (81.5)	122 (50.4)	396 (68.5)	09 (2.9)	
Yes	62 (18.5)	120 (49.6)	182 (31.5)	08 (5.2)	
**Mother support**^b^					0.239
No	23 (6.8)	32 (13.2)	55 (9.5)	00 (0.0)	
Yes	313 (93.2)	210 (86.8)	523 (90.5)	17 (4.0)	
**Pregnancy planning**^b^					0.069
No	89 (26.5)	122 (50.4)	211 (36.5)	10 (5.9)	
Yes	247 (73.5)	120 (49.6)	367 (63.5)	07 (2.3)	
**Subsyndromic depression (BDI-II)**^b^					0.008
No	284 (84.5)	86 (35.5)	370 (64.0)	05 (1.7)	
Yes	52 (15.5)	156 (64.5)	208 (36.0)	12 (7.6)	
**Subsyndromic anxiety (BAI)**^b^					0.765
No	307 (91.4)	158 (65.3)	465 (80.4)	13 (3.5)	
Yes	29 (8.6)	84 (34.7)	113 (19.6)	04 (4.0)	
**Stressful events (EARS-Homes/Rahe)**^a^^b^					0.082
No	222 (66.3)	49 (20.2)	271 (47.0)	04 (1.9)	
Yes	113 (33.7)	193 (79.8)	306 (53.0)	13 (5.1)	
**History of chronic illness**^b^					0.505
No	298 (88.7)	174 (71.9)	472 (81.7)	13 (3.3)	
Yes	38 (11.3)	68 (28.1)	106 (18.3)	04 (5.1)	
**History of depression**^b^					0.721
No	324 (96.4)	180 (74.4)	504 (87.2)	14 (3.5)	
Yes	12 (3.6)	62 (26.6)	74 (12.8)	03 (4.5)	

					
**Intervention**					0.079
No (control)	-	-	336 (59.3)	06 (2.2)	
Yes (risk)	-	-	242 (40.7)	11 (5.5)	
**Total**	336 (100.0)	242 (100.0)	578 (100.0)	17 (3.6)	

a variable with missing.

b variables that presented p < 0.05 between control and risk groups in x^2^.

The variables that present p < 0.20 in the bivariate analysis (Table 1) were taken to a *Poisson* regression-adjusted analysis: therapy model (control/risk for depression), age, schooling, living with a partner, pregnancy planning, subsyndromal depression and stressor events. The variable of the Z-score of the sum of risk criteria was used for separate analyses and, therefore, did not cause any confusion with the results. After adjusting for confounding factors, the results continued to show PPD remained associated with women of 30 years of age or older and with less schooling (Table 2).

**Table 2:** Poisson regression multivariate analysis.

	**POST-PARTUM DEPRESSION**	
	**RP (95% CI)**	**p-value**
**Age**		
Up to 23 years	1	-
Between 24 and 29 years	1.49 (0.27-0.08)	0.645
30 years or more	5.13 (1.28-20.48)	0.021
**Schooling**		
9 years of study or more	1	-
Up to 8 years of study (lower quartile)	4.12 (1.38-12.33)	0.011
**Intervention**		
No (control)	1	-
Yes (risk)	1.66 (0.44-6.18)	0.451

adjusted for age, schooling, living with a partner, pregnancy planning, subsyndromal depression, stressful events and therapy model (intervention).

Following these analyses, an evaluation of the impact of the therapeutic process was performed through the OQ-45 instrument and applied to all women in the risk group and the group that received preventive psychotherapy.

**Fig. 2:**
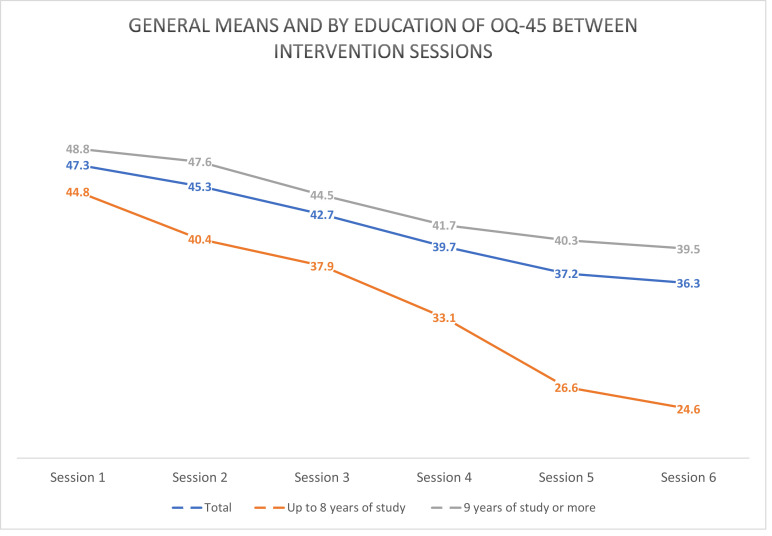
Means of OQ-45 between therapy sessions.

The Fig. 2 shows the averages of the self-perception of improvement for each session. It is observed that the overall average in the first session was 47.3 points, while in the last session of the protocol (session 6) the average was 36.3 points. These values gradually decreased over the course of the therapy. When considering the difference in the means in the general sample between the first and sixth session, we found that there was a significant reduction in emotional distress assessed by the OQ-45 (p < 0.01).

When comparing the self-perception of improvement separated by schooling, we verified a greater level of therapeutic progress (OQ-45) among pregnant women with less schooling. In the values of the means of the lower quartile groups of schooling *versus* the others, it is observed that already in the second session there was a difference that is accentuated until the end of psychotherapy (p < 0.01) (Fig. 2).

As an additional analysis, the severity of depressive symptoms was also explored in order to add to the better understanding of the results, with an emphasis on schooling. Regarding the age variable, no difference was found in the means of depressive symptoms in the postpartum period (T3) in relation to baseline (T1), between the control and risk groups, and no differences were found regarding the means of the OQ-45 (p > 0.05).

**Fig. 3:**
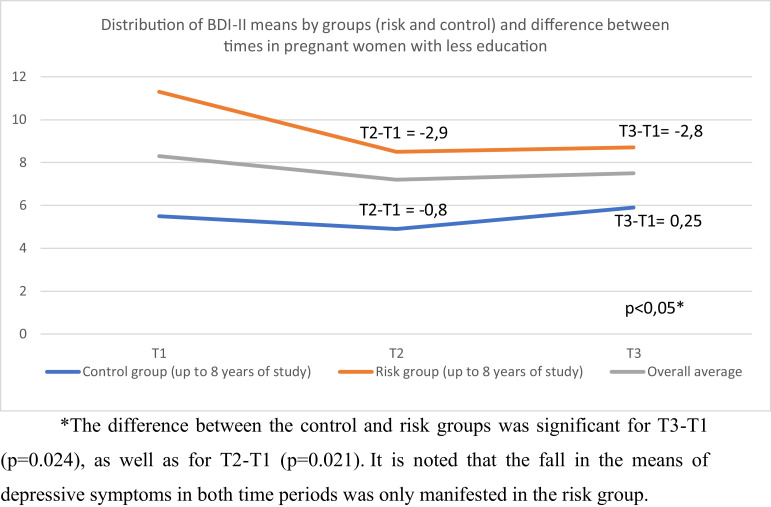
Distribution of BDI-II averages between evaluations.

The Fig. 3 shows the means of the BDI-II and their differences between the times of the evaluations in pregnant women with less schooling (up to 8 years of schooling/lower quartile), comparing control and risk for depression.

In the group with higher schooling, there was no difference in the means of the BDI-II of T3-T1 between the risk and control groups, indicating that the group with higher schooling remained with depressive symptomatology of equal intensity, regardless of the therapy.

When we moved on to an analysis of the factor of less schooling, we found a prevalence of PPD of 7.1% for the risk group and 9.1% for the control group (p = 0.732), with no association between control and risk in the manifestation of PPD (PR 0.77 95% CI 0.19-3.03). However, when we separately evaluated the group with higher schooling, we found a prevalence of PPD of 4.7% in the risk group and 0.9% in the control group, and women in the risk group and with higher schooling have 5.46 (95% CI 1.09-27.48) times higher PPD when compared to women in the control group.

## Discussion

The results of this study indicate the effectiveness of a preventive cognitive behavioral therapy for PPD. Of all the women evaluated at risk of developing the disorder, 5.5% presented the diagnosis in an evaluation performed 90 days after delivery. In addition, we found that the manifestation of depressive conditions was no longer associated with the risk group when compared to a control group, because, although there was a percentage difference in the initial analysis, this was not significant, and did not remain associated with the results after multivariate analysis. This finding indicates that the therapy group and the control group behaved similarly in relation to the presence of PPD.

By performing a preventive therapy based on previously studied risk factors, we created a condition of greater exposure. For this reason, we selected in advance in the bibliography the ten factors most associated with PPD, and many of these factors have a strong association with the disorder. The prevalence found, of 5.5% does not necessarily mean that we prevented 94.5% of women from falling ill postpartum, however, it is significant that compared to women who were not at high risk, the evolution of PPD was similar. Thus, we can assume that this therapy was effective in preventing PPD.

Following the same train of thought, we verified an association between the z score of the sum of risk criteria and the presence of PPD, which reinforces the association between these characteristics and the manifestation of the disease. However, when we analyzed the control and risk groups separately, no association was found, which indicates that preventive treatment was able to reduce the impact of risk factors on the PPD results, equaling the manifestation of the disorder between the control groups and risk of depression groups.

An element also proposed in the analysis, was the therapeutic progress observed throughout the sessions. It was possible to detect a gradual decrease in the means of the OQ-45, which indicates that the pregnant women identified a decrease in emotional distress related to the issues focused on the preventive therapy model, even without a previous diagnosis of depression. It is believed that the fact of addressing dysfunctional thoughts related to pregnancy and postpartum, offering a new perspective to patients on what is and what will be experienced could bring the perception of improvement, reflecting in a low incidence of PPD also in this group. We should remember that this group was selected because it had three or more high risk factors for PPD.

However, one factor in particular led us to focus on more specific analyses: schooling. When we obtained a significant decrease in depressive symptoms in the risk group in relation to the control group, particularly with less schooling, we can infer that our therapy was more effective in this subgroup. This finding raises three questions: less schooling alone should be considered a possible factor that indicates the need for special attention in the pregnancy-puerperal cycle, even in those women who do not present cumulative risk factors; our therapy obtained a better symptom response to depression in women at risk of PPD with less schooling; and, finally, that the model of our therapy protocol may be more appropriate for women with less schooling.

The fact that lower levels of schooling is associated with PPD is mentioned in studies (Matsumura et al., 2019; Santos et al., 2021), and should continue to receive attention. In the control group those with lower levels of schooling evolved more frequently to PPD. Although this condition is a risk factor, we found that the women who participated in the preventive therapy responded both with greater therapeutic progress and by the reduction of depressive symptoms. Moreover, although the prevalence of PPD is higher in the risk group compared to the control group, this difference was not significant when we observed only those with less schooling.

When we proposed this therapy, our secondary objective was to test the hypothesis that therapists with basic training and with the training we provided, along with the monitoring by professionals with specific training in CBT, would be able to effectively perform a preventive therapy protocol in CBT early in the pregnancy. Thus, all psychotherapists had basic training, so that once the therapy was proven effective, we could expand to other centers, with results indicating that it is not essentially necessary to have specialized training to provide effective execution of this protocol. This would certainly lower costs and enable a labor force without specialization in the area to perform such therapy as long as it followed a structured protocol, supervised by a trained professional. Therefore greatly increasing the application of this therapy model. This was based on the idea that such a model, once proven effective, would become viable for use in basic public health care.

One limitation of our study is the rate of refusal to preventive treatment, which was 40.5% of the initial referrals. However, non-acceptance rates (refusal) to this type of therapy are also high in other samples, which may indicate that 59.5% is a reasonable acceptance rate. When we established a z score of the sum of the risk criteria, there was no difference between the means of those who refused and those who agreed to start the therapy, so the risk load was not different between the groups. Another limitation was the losses during the therapeutic process, in the control group 19.7% and 17.8% in the risk group did not attend the postpartum follow-up.

In addition, it is worth mentioning that these pregnant women had no indication of treatment for any diagnosis, but criteria considered at risk for developing depression, which may have made it difficult to understand the importance of the suggested follow-up, as well as in the motivation that a pregnant woman who proposes to attend six meetings at a pre-scheduled time and place, taking her away from her daily activities in the midst of a pregnancy. Austin (2008), in a cut-out on risk for depression, cites that pregnant women with subsyndromal depressive symptoms may reject the notion that they may have postpartum problems (Austin et al., 2008).

The low prevalence of PPD compared to other studies using screening instruments to measure such results could indicate one more limitation (Levis et al., 2019). However, the choice of a diagnostic instrument such as Mini Plus was in line with the objectives of this therapy study, aiming at a diagnosis and not a screening. Mini Plus is an interview adapted to a clinical context and the evaluation of more severe patients, and represents an economic alternative for the selection of patients, according to international criteria, both in clinical and epidemiological studies (Amorim, 2000). The evaluation was made by interviewers with previous training, periodic reinforcements and review of each interview with positive diagnosis. In addition, we used for the severity of symptoms the BDI-II, a widely used measure that can be parameterized for lifelong measurements, thus creating comparable measures at different times.

We performed multiple analyses as shown in the results session. In the case of the main risk factor associated with the results, level of schooling, when calculating the power of the sample, found a power of 79.3%. This strengthens the results, both of the greater effectiveness of the therapy in those with less schooling, measured by Mini Plus and BDI-II with regard to depressive symptomatology, and by the perception of improvement measured by the OQ-45. This should be considered positively and not as a limitation of the results. Also, the absence of a group with similar characteristics in another therapy model may have been a limitation of our study, and a control group without therapy is the comparative group. However, the choice of therapy was made by the model with strong indications of efficacy against depression.

In this sequence, it is also worth mentioning as a virtue of our study the psychotherapy offered individually, which may be more effective than group psychotherapy for pregnant women at risk of developing PPD, as suggested by Austin et al. (2008).

Given the above, by our findings we understand that testing protocols that can prevent PPD, alleviating the psychological suffering of mothers during this period and avoiding long-term losses, remains extremely relevant. This is due, both because PPD is often the first episode of suffering due to mood disorder that will be repeated during life, bringing significant disability to this woman and negative consequences for her offspring, and for reducing the impact of the high costs that such a disease causes to health and social security systems, public and private, throughout the life of this woman. Such emotional and financial repercussions are repeatedly reported in studies on perinatal complications due to depression in the puerperal pregnancy cycle (de Menezes et al., 2012).

We evaluated that this impact can be reduced with the adoption of an effective protocol. With regard to the public health system, a protocol similar to that applied by our study (brief and consequently low cost, effective in preventing the manifestation of PPD, especially among women with less schooling and which can be applied by mental health professionals with basic education) has the potential to positively impact patients. Finally, we consider that this study should be replicated in other places, especially with socioeconomically vulnerable populations, with due sociocultural adaptation of the protocol. Thus, we can obtain a wider external validity and thereby strengthen the case of this therapy proposal.

## Author Statement Contributors

R. T. Pinheiro is principal investigator and project manager. M. de Matos, L. Quevedo, K. Pinheiro, J. Motta, F. Nedel, A. Ardais G. Ghisleni, F. Coelho and R. Silva authors contributed to the conception and design of the study and participated in the elaboration of the questionnaire. M. de Matos, C. Martins, G. da Cunha, R. Stigger, F. Coelho and J. Trettim coordinated the fieldwork and participated in the acquisition of data. R. T. Pinheiro, J. Trettim and L. Souza contributed to the writing of the article and inclusion of articles in the review. All authors provided feedback on drafts of the manuscript and interpreted the results and all authors have approved the final manuscript.

## Declaration of Competing Interest

The authors declare that they have no conflict of interest.
